# Sex-dependent effects of acute stress and alcohol exposure during adolescence on mRNA expression of brain signaling systems involved in reward and stress responses in young adult rats

**DOI:** 10.1186/s13293-024-00649-5

**Published:** 2024-09-26

**Authors:** Carlotta Gobbi, Laura Sánchez-Marín, María Flores-López, Dina Medina-Vera, Francisco Javier Pavón-Morón, Fernando Rodríguez de Fonseca, Antonia Serrano

**Affiliations:** 1 Instituto de Investigación Biomédica de Málaga y Plataforma en Nanomedicina (IBIMA- Plataforma BIONAND), Málaga, 29590 Spain; 2 Unidad de Gestión Clínica de Salud Mental, Hospital Regional Universitario de Málaga, Málaga, 29010 Spain; 3 Unidad Clínica Área del Corazón, Hospital Universitario Virgen de la Victoria de Málaga, Málaga, 29010 Spain; 4 Centro de Investigación Biomédica en Red de Enfermedades Cardiovasculares (CIBERCV), Instituto de Salud Carlos III, Madrid, 28029 Spain; 5 Unidad de Gestión Clínica de Neurología, Hospital Regional Universitario de Málaga, Málaga, 29010 Spain

**Keywords:** Binge drinking, Restraint, Adolescence, Sexual dimorphism, HPA axis, Amygdala, Hypothalamus

## Abstract

**Background:**

Adolescent stress and alcohol exposure increase the risk of maladaptive behaviors and mental disorders in adulthood, with distinct sex-specific differences. Understanding the mechanisms underlying these early events is crucial for developing targeted prevention and treatment strategies.

**Methods:**

Male and female Wistar rats were exposed to acute restraint stress and intermittent alcohol during adolescence. We assessed lasting effects on plasma corticosterone (CORT) and adrenocorticotropic hormone (ACTH) levels, and mRNA expression of genes related to corticotropin releasing hormone (CRH), neuropeptide Y (NPY), corticoid, opioid, and arginine vasopressin systems in the amygdala and hypothalamus.

**Results:**

The main findings are as follows: (1) blood alcohol concentrations (BAC) increased after the final alcohol administration, but stressed males had lower BAC than non-stressed males; (2) Males gained significantly more weight than females; (3) Stressed females showed higher ACTH levels than non-stressed females, with no changes in males; (4) Stress increased CORT levels in males, while stressed, alcohol-treated females had lower CORT levels than non-stressed females; (5) CRH: Females had lower *Crhr1* levels in the amygdala, while alcohol reduced *Crhr2* levels in males but not females. Significant interactions among sex, stress, and alcohol were found in the hypothalamus, with distinct patterns between sexes; (6) NPY: In the amygdala, stress reduced *Npy* and *Npy1r* levels in males but increased them in females. Alcohol decreased *Npy2r* levels in males, with varied effects in females. Similar sex-specific patterns were observed in the hypothalamus; (7) Corticoid system: Stress and alcohol had complex, sex-dependent effects on *Pomc*, *Nr3c1*, and *Nr3c2* in both brain regions; (8) Opioid receptors: Stress and alcohol blunted the elevated expression of *Oprm1*, *Oprd1*, and *Oprk1* in the amygdala of males and the hypothalamus of females; (8) Vasopressin: Stress and alcohol interacted significantly to affect *Avp* and *Avpr1a* expression in the amygdala, with stronger effects in females. In the hypothalamus, alcohol increased *Avp* levels in females.

**Conclusions:**

This study demonstrates that adolescent acute stress and alcohol exposure induce lasting, sex-specific alterations in systems involved in reward and stress responses. These findings emphasize the importance of considering sex differences in the prevention and management of HPA dysfunction and psychiatric disorders.

**Supplementary Information:**

The online version contains supplementary material available at 10.1186/s13293-024-00649-5.

## Abstract (plain language summary)

Experiencing stress and consuming alcohol during the teenage years can significantly raise the risk of developing mental health problems later in life. These risks can vary between males and females, making it important to understand how each sex is differently affected. This study investigated how exposure to acute stress and intermittent alcohol use during adolescence impacts male and female rats as they reach young adulthood. Our research involved first measuring the levels of stress hormones in the blood to see how these early experiences influenced the body’s stress response. Following this, we closely examined changes in the expression of specific genes within two critical brain regions: the amygdala and the hypothalamus. These regions play a crucial role in controlling emotional states, managing stress, and regulating behaviors related to addiction. The results of the study revealed that both stress and alcohol exposure during adolescence led to lasting alterations in stress hormone levels and changes in the expression of genes within the brain. Importantly, these effects were not the same for males and females, highlighting the presence of sex-specific differences. Understanding these differences can guide the development of more effective treatments, ultimately improving mental health outcomes for both males and females.

**Supplementary Information:**

The online version contains supplementary material available at 10.1186/s13293-024-00649-5.

## Highlights

Acute stress led to decreased BAC levels in male rats after the final alcohol administration in a binge drinking model, without effects on female rats.

Stressed female rats showed elevated ACTH levels compared to non-stressed females, while interactions between stress and alcohol produced sex-specific effects on CORT levels.

Adolescent alcohol increased *Crh* gene expression in the amygdala in both sexes, with additional sex-dependent alterations in related receptor genes in the amygdala and hypothalamus.

Stress and alcohol had sexually dimorphic effects on *Npy* gene expression in the amygdala, lowering levels in stressed males but increasing them in stressed females.

The expression of opioid receptor genes, such as *Oprm1* and *Oprd1*, was significantly altered by adolescent stress and alcohol exposure, with sex-specific patterns in the amygdala and hypothalamus.

**Supplementary Information:**

The online version contains supplementary material available at 10.1186/s13293-024-00649-5.

## Background

Adolescence is a dynamic period of brain development characterized by significant structural and functional changes, particularly in regions associated with impulse control, decision-making, and emotional regulation. These neurodevelopmental changes are pivotal in shaping the behavioral, emotional, and cognitive alterations characteristic of this stage. Additionally, many of these developmental processes exhibit sex-specific patterns, primarily influenced by gonadal hormones [1].

Alterations in both brain structure and function during adolescence can increase susceptibility to the harmful effects of alcohol consumption. Notably, alcohol use at this critical stage is recognized as a significant risk factor for the later development of psychiatric disorders, including alcohol use disorder (AUD) and mood disorders [2, 3]. Furthermore, extensive research has demonstrated that alcohol exposure during adolescence leads to substantial transcriptional changes in key brain regions through epigenetic modifications, impacting genes involved in neuroplasticity, inflammation, and neurotransmission, which are critical for understanding the resultant behavioral phenotypes [for review see [4–9]]. For instance, alcohol use during adolescence results in a long-term reduction in *Arc* expression − an immediate early gene product essential for regulating neuronal function and synaptic plasticity in the amygdala with behavioral consequences − by suppressing *Arc* enhancer RNA expression [10]. Interestingly, restoring *Arc* expression through targeted epigenomic editing reversed the effects of adolescent alcohol consumption on excessive drinking and anxiety-like behavior in adulthood [11]. Although adolescents may not drink as frequently as adults, they often engage in binge drinking, defined as consuming at least five drinks for males or four drinks for females within a 2-hour period, leading to a blood alcohol concentration (BAC) of 80 mg/dL or higher [12]. Notably, sexual dimorphism in the behavioral, health, and social outcomes of binge drinking has important implications for both treatment and prevention strategies [13]. Previous preclinical research has shown that while some alcohol-related effects are consistent across sexes, others vary significantly between males and females due to a combination of biological, physiological, and hormonal factors. This variability highlights the critical importance of considering sex as a biological variable when studying the effects of adolescent alcohol exposure [for review see [14]].

In addition to alcohol consumption, exposure to stressful episodes during adolescence can lead to lasting behavioral effects, including increased vulnerability to the early onset of problematic drinking and the development of psychiatric disorders, such as AUD and anxiety in adulthood [15–18]. The stress response engages and impacts a wide range of brain structures involved in stress coping, emotional regulation, cognitive function, and learning and memory, including the prefrontal cortex, amygdala, hippocampus, hypothalamus, and nucleus accumbens [19]. The impact of stress at each developmental stage is partly dependent on the maturational state of these brain regions at the time of exposure, influencing factors such as the rate of neurogenesis, synaptic pruning, and the expression of glucocorticoid and mineralocorticoid receptors. Adolescence is characterized by increased stress and heightened stress reactivity compared to adulthood [20]. For instance, the hypothalamic-pituitary-adrenal (HPA) axis undergoes significant changes during the transition to adolescence, both in basal activity and in response to stressors. In adolescent animals, the HPA axis response to acute stress is exaggerated and highly plastic, with a delayed return to basal levels following stress exposure [21]. Changes in the expression of immediate early genes (e.g., *c-Fos*, *Egr1*, and *Arc*), neuroimmune genes (e.g., *Il1b*), and glial markers (e.g., *Cd14*) associated with the HPA axis have been studied in stress-related regions such as amygdala, hippocampus, and hypothalamus, in rats exposed to stress during adolescence, showing an exacerbation in the expression of these genes, accompanied by increased emotional reactivity [22–24]. Similar to alcohol exposure, there are sex differences in stress sensitivity, with sex-specific vulnerabilities to stress-induced psychiatric disorders, including major depressive disorder and anxiety disorders [25, 26]. Previous studies in animal models have demonstrated that females exhibit greater vulnerability to the effects of early stress [27, 28].

Therefore, both stress and alcohol consumption during vulnerable developmental periods have lasting detrimental effects on the brain and behavior, which may occur synergistically or independently. In this regard, we have previously demonstrated that both restraint stress and alcohol binge drinking during adolescence lead to persistent anxiety-like behaviors, although distinct mechanisms are involved in these maladaptive changes within the brain [29, 30]. However, these findings are limited as they were conducted only in male rats, which precludes the investigation of sexual dimorphism in these molecular and behavioral alterations.

The present study aims to investigate how stress and alcohol exposure during adolescence affect the gene expression of signaling systems involved in the stress response and the modulation of emotion and reward, with a focus on sex differences. We hypothesize that these molecular systems exhibit specific, lasting alterations in response to stress and/or alcohol, which are sex-dependent and may contribute to maladaptive behaviors and increased susceptibility to psychiatric disorders. To test this hypothesis, adolescent male and female Wistar rats were subjected to an acute restraint stress episode and/or intermittent alcohol exposure using a binge-like procedure. Specifically, we measured the mRNA expression of genes associated with signaling systems related to corticotropin-releasing hormone (CRH), neuropeptide Y (NPY), opioid receptors, and vasopressin in both the amygdala and hypothalamus. The amygdala was selected due to its involvement in the regulation of anxiety and emotion, along with its role in the modulation of reward and addiction-related behaviors [31]. The hypothalamus was chosen for its critical role in the negative feedback control of the HPA axis and regulation of the stress response [32]. Given that the HPA axis is a key regulatory mechanism in the stress response, and its dysregulation may contribute to the interaction between adolescent stress and the development of psychiatric disorders later in life [33, 34], we also examined HPA axis activity in these animals. To this end, we also assessed plasma levels of adrenocorticotropic hormone (ACTH) and corticosterone (CORT), as well as the gene expression of the ACTH precursor proopiomelanocortin (POMC) and the glucocorticoid and mineralocorticoid receptors in these brain regions. All these assessments in different signaling systems involved in reward and stress responses may provide an integrative understanding of how molecular alterations, with a significant influence of sex, could have a long-term impact on adult behavior and the risk of psychiatric disorders.

## Methods

### Animals and ethics statement

Forty-eight Wistar rats, 24 male rats and 24 female rats, with a weight range of 75–100 g, were acquired from Charles River Laboratories (France) on postnatal day 20 (PND20). Upon arrival, the rats were paired and placed in a vivarium with controlled humidity and temperature on a 12-hour light/dark cycle with lights turned off at 19:00 h. An acclimatization period to the new environment was allowed before initiating any experimental procedures on PND29. Throughout this period and the course of the study, rats had unrestricted access to water and standard rat chow pellets (Ref. LASQCdiet Rod14, Altromin, Lage, Germany).

This study was designed and conducted in adherence to the European directive 2010/63/EU for the protection of animals utilized for scientific purposes. Additionally, it conformed to the Spanish regulations for the care and use of laboratory animals (Real Decreto 53/2013 and 178/2004, Ley 32/2007 and 9/2003, and Decreto 320/2010). All protocols and procedures underwent approval by the Ethic and Research Committee of the Universidad de Málaga (CEUMA) in accordance with the ARRIVE (Animal Research: Reporting of In Vivo Experiments) guidelines [35]. All efforts were undertaken to minimize any potential suffering experienced by the animals and to reduce the number of animals involved in the study.

### Experimental design: acute restraint stress and intermittent alcohol procedures

Rats of both sexes were randomly assigned to the experimental subgroups: male rats [non-stressed saline-treated (*n* = 6), stressed saline-treated (*n* = 6), non-stressed alcohol-treated (*n* = 6), and stressed alcohol-treated (*n* = 6) rats] and female rats [non-stressed saline-treated (*n* = 6), stressed saline-treated (*n* = 6), non-stressed alcohol-treated (*n* = 6), and stressed alcohol-treated (*n* = 6) rats].

On PND28, a group of 12 male and 12 female rats underwent a single 90-minute session of restraint stress, as previously described [30]. On the contrary, the rats in the non-stress subgroup (*n* = 12 male and *n* = 12 female rats) were allowed to remain undisturbed in their home-cages. Then, rats of both sexes received repeated intragastric ethanol administrations via gavage from PND31 to PND55, as previously described [29, 30]. Briefly, rats from both stress (*n* = 6 male and *n* = 6 female rats) and non-stress (*n* = 6 male and *n* = 6 female rats) subgroups received 3 g/kg of ethanol in a volume of 15 mL/kg (25% ethanol in saline, v/v) over 4 consecutive days, followed by 3 days of alcohol deprivation, repeated for 4 weeks. Control rats in the stress (*n* = 6 male and *n* = 6 female rats) and non-stress (*n* = 6 male and *n* = 6 female rats) subgroups were administered an isovolumetric equivalent of saline following the same schedule and procedure. All the animals were weighed every week.

### Determination of blood alcohol concentration

Blood alcohol concentration (BAC) levels were measured for each rat in the alcohol subgroups that underwent tail bleeding 1 h after the initial (PND31) and final (PND55) alcohol administration during intermittent alcohol exposure. Blood samples were collected and then centrifuged at 2000 ×g for 15 min to obtain plasma. The plasma samples were analyzed for BAC using the alcohol oxidase method with an AM1 Alcohol Analyzer (Analox Instruments, London, UK). After the adolescent alcohol/saline exposure, the rats were left undisturbed in their home-cages for 2 weeks.

### Sample collection

Two weeks after the final alcohol administration (PND70), rats were anesthetized with sodium pentobarbital (50 mg/kg, i.p.), and blood and brain samples were collected for subsequent processing.

#### Blood extraction and processing

Blood samples were obtained via trunk blood collection following the administration of the anesthetic, using 9 mL blood collection tubes with K_2_EDTA. These tubes containing blood were immediately centrifuged at 2000 ×g for 15 min, and a series of plasma aliquots were preserved for subsequent analysis.

#### Brain dissection

Parallel to blood extraction, brain samples were collected and rapidly frozen on dry ice, then stored at -80 ºC until molecular analyses. For these analyses, the frozen brains were dissected using acrylic rat brain matrices. Two-mm thick slices were obtained using razor blades specifically designed for the brain matrix. Subsequently, the amygdala and hypothalamus were bilaterally dissected and collected using a sample corer. The precise localization of these brain regions was determined using a rat brain atlas [36].

### Immunoassay-based quantification of plasma ACTH and CORT levels

Commercially available enzyme-linked immunoassay (ELISA) kits were used to assess plasma ACTH (ab263880, Abcam Limited, Cambridge, UK) and CORT (ab108821, Abcam Limited, Cambridge, UK) levels, according to the manufacturer’s instructions.

### RNA isolation and RT-qPCR analysis of brain samples

Real-time PCR was employed to quantify the relative mRNA levels of several neuropeptides and receptors involved in reward and stress responses: Corticotropin-releasing hormone (CRH, *Crh*) and CRH receptors [CRHR1 (*Crhr1*) and CRHR2 (*Crhr2*)]; Neuropeptide Y (NPY, *Npy*) and NPY receptors [NPY1r (*Npy1r*) and NPY2r (*Npy2r*)]; Proopiomelanocortin (POMC, *Pomc*); Corticoid receptors [Glucocorticoid receptor (*Nr3c1*) and mineralocorticoid receptor (*Nr3c2*)]; Opioid receptors [Mu-opioid receptor (MOR, *Oprm1*), kappa-opioid receptor (KOR, *Oprk1*), and delta-opioid receptor (DOR, *Oprd1*)]; Sigma-1 receptor (S1R, *Oprs1*); Opioid-related nociception receptor 1 (NOR, *Oprl1*); and Arginine vasopressin (AVP, *Avp*) and AVP receptor (AVPR1a, *Avpr1a*). RT-qPCR was conducted as previously described [29], using TaqMan Gene Expression Assays (Thermo Fisher Scientific, Waltham, MA, USA) with specific primer sets. Primer sequences were obtained from the genome database of all TaqMan Assays for mRNA analysis in [Rn] rat. (**Table ****S1**).

Total RNA was extracted from brain samples using Trizol Reagent (Gibco BRL Life Technologies, Baltimore, MD, USA), and RNA concentrations were measured with a spectrophotometer to ensure absorbance ratios at 260/280 nm were within the acceptable range of 1.8–2.0. Reverse transcription was carried out using the Transcriptor Reverse Transcriptase kit and random hexamer primers (Transcriptor RT; Roche Diagnostic, Mannheim, Germany). Subsequent RT-qPCR was performed on an ABI PRISMR 7300 Real-Time PCR System (Applied Biosystems, Foster City, CA, USA) using the FAM dye label format for the TaqMan Gene Expression Assays (Applied Biosystems, Foster City, CA, USA). Absolute values from each sample were normalized to the endogenous control genes beta-actin (*Actb*) and beta-2 microglobulin (*β2m*). Relative quantification was calculated using the ΔΔCt method and normalized to the control group.

### Statistical analysis

All data are expressed as the mean ± standard error of the mean (SEM). The normality of the data distribution was assessed using the D’Agostino-Pearson omnibus test. The significance of differences within and between subgroups was evaluated using three-way analysis of variance (ANOVA) with ‘sex’ (F_1_, levels: ‘male’ and ‘female’), ‘stress’ (F_2_, levels: ‘non-stress’ and ‘stress’), and ‘alcohol’ (F_3_, levels: ‘saline’ and ‘alcohol’) as factors. In cases where significant three-way interactions were identified, separate two-way ANOVAs were conducted for male and female rats, with ‘stress’ and ‘alcohol’ as factors. *Post hoc* multiple comparisons were performed using Tukey’s test when a significant interaction was identified by the ANOVA.

Test statistic values (*F*-values) and degrees of freedom are reported in the results. A *p*-value of less than 0.05 was considered statistically significant. All statistical analyses were performed using Graph-Pad Prism version 5.04 software (GraphPad Software, San Diego, CA, USA).

## Results

### Effects of acute stress, alcohol administration timing, and sex on BAC in rats

**Fig. 1:**
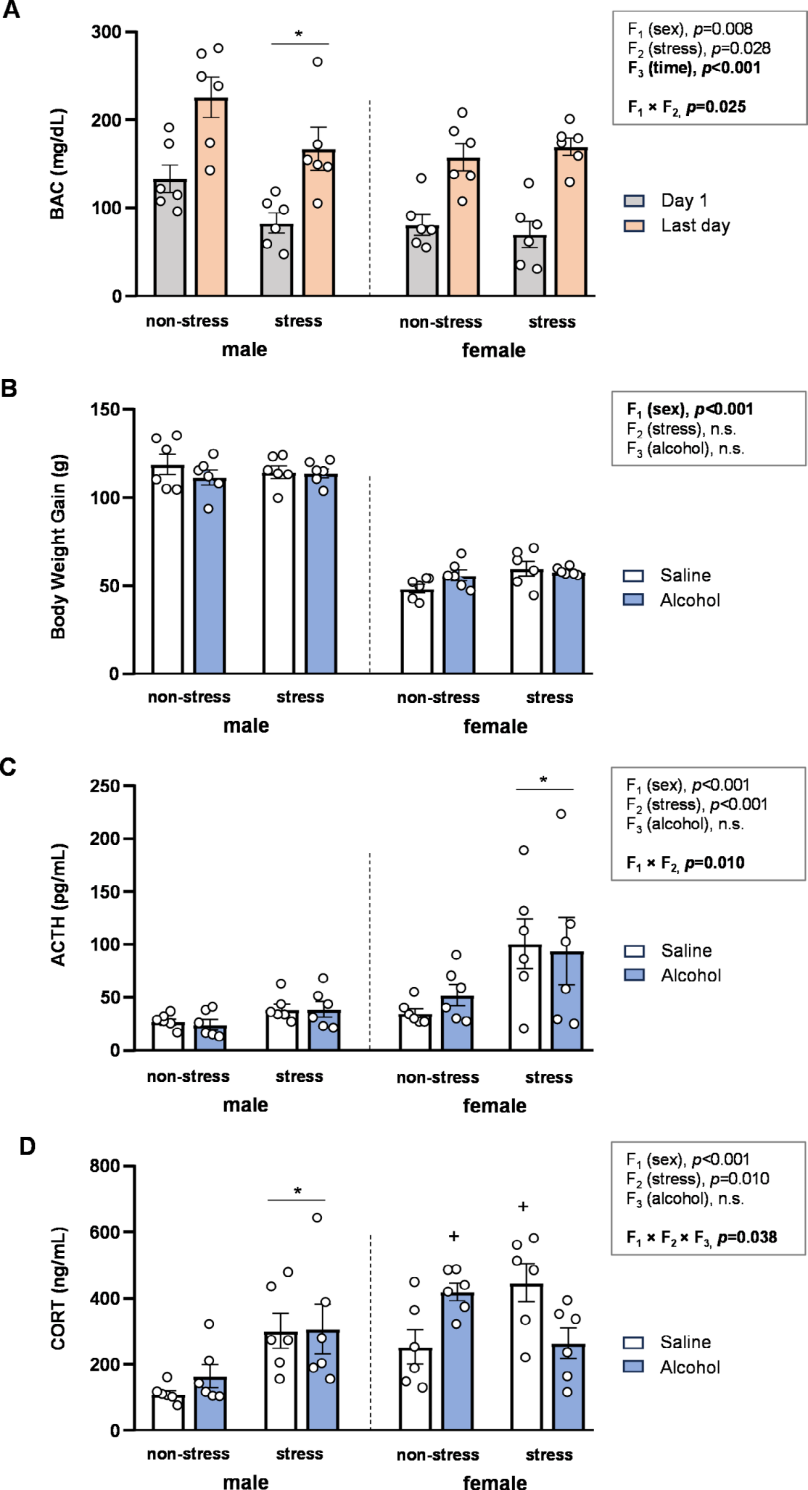
**Effects of acute stress**,** adolescent alcohol**,** and sex on BAC**,** body weight gain**,** and plasma ACTH and CORT levels.** BAC at two different time-points of the intermittent alcohol exposure **(A)**; total body weight gain **(B)**; plasma ACTH levels **(C)**; and plasma CORT levels **(D)** in male and female rats exposed to acute stress and alcohol exposure during adolescence. Bars represent the mean ± SEM (6 rats/subgroup). Data were analyzed using three-way ANOVA. Symbols are used to represent how significant interactions occur: (*) denotes *p* < 0.05, comparing stressed rats to non-stressed rats in males or females; (+) denotes *p* < 0.05, comparing rats to non-stressed, saline-treated rats in males or females

BAC levels were measured in plasma samples from rats of both sexes at two different time points, after the first and after the last administration of alcohol. We conducted a three-way ANOVA with the following factors: sex (F_1_), stress (F_2_), and time (F_3_). As shown in Fig. 1A, the analysis revealed a significant main effect of time [*F* [1,40] = 56.7, *p* < 0.001], indicating that BAC levels were higher after the final alcohol administration compared to the first day in both sexes. Additionally, a significant interaction between sex and stress [*F* [1,40] = 5.46, *p* = 0.025] was observed, where stressed male rats exhibited lower BAC levels than non-stressed male rats, an effect not seen in female rats.

### Effects of acute stress, adolescent alcohol, and sex on body weight gain in rats

Another three-way ANOVA with sex (F_1_), stress (F_2_), and alcohol (F_3_) as factors was conducted to evaluate the effects and interactions of these factors on body weight gain (Fig. 1B). The analysis revealed only a significant main effect of sex [*F* [1,40] = 527.5, *p* < 0.001], showing that weight gain was greater in male rats compared to female rats.

### Effects of acute stress, adolescent alcohol, and sex on stress-related hormonal levels in rats

We assessed the effects of acute stress and adolescent alcohol exposure on plasma ACTH and CORT levels in male and female rats. Sex (F_1_), stress (F_2_), and alcohol (F_3_) were considered as factors in the statistical analysis.

#### Plasma ACTH levels

As shown in Fig. 1C, while there were main effects of sex and stress on ACTH levels, a significant interaction between both factors was also found [*F* [1,40] = 7.33, *p* = 0.010]. Specifically, stressed female rats exhibited higher ACTH levels compared to non-stressed female rats; an effect not observed in male rats.

#### Plasma CORT levels

Regarding CORT levels, the analysis revealed main effects of sex and stress, along with a significant three-way interaction involving sex, stress, and alcohol [*F* [1,40] = 4.62, *p* = 0.038] (Fig. 1D).

To better understand the interactions between stress and alcohol, we analyzed the data separately by sex using two-way ANOVAs: (a) In male rats, there was a significant main effect of stress [*F* [1,20] = 11.59, *p* = 0.003], with stressed rats displaying higher CORT levels than non-stressed rats. (b) However, a significant interaction between stress and alcohol was observed in female rats [*F* [1,20] = 13.34, *p* = 0.002]. *Post hoc* comparisons revealed that stressed saline-treated rats had significantly higher CORT levels compared to non-stressed saline-treated female rats (*p* < 0.05). Additionally, alcohol-treated rats exhibited significantly lower CORT levels compared to saline-treated female rats within the stress subgroup (*p* < 0.05).

### Effects of acute stress, adolescent alcohol, and sex on mRNA expression of CRH signaling-related genes in the brains of rats

**Fig. 2:**
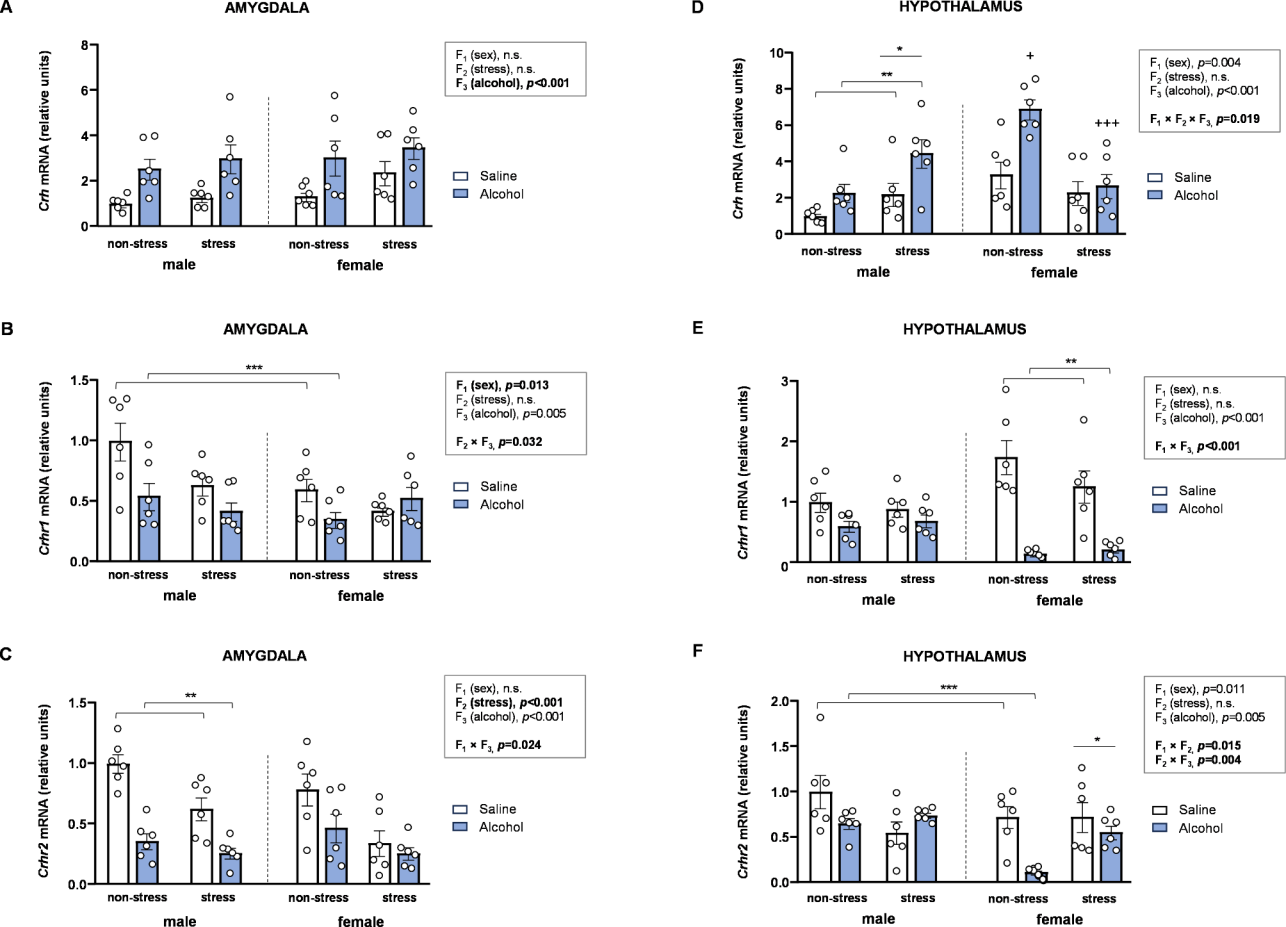
**Effects of acute stress**,** adolescent alcohol**,** and sex on CRH signaling-related genes in the amygdala and hypothalamus. ***Crh* expression in the amygdala **(A)**; *Crhr1* expression in the amygdala **(B)**; *Crhr2* expression in the amygdala **(C)**; *Crh* expression in the hypothalamus **(D)**; *Crhr1* expression in the hypothalamus **(E)**; and *Crhr2* expression in the hypothalamus **(F)** of male and female rats exposed to acute stress and alcohol exposure during adolescence. Bars represent the mean ± SEM (6 rats/subgroup). Data were analyzed using three-way ANOVA. Symbols are used to represent how significant interactions occur: (*) denotes *p* < 0.05, comparing stressed rats to non-stressed rats in males or females; (**) denotes *p* < 0.05, comparing alcohol-treated rats to saline-treated rats in males or females; (***) denotes *p* < 0.05, comparing alcohol-treated rats to saline-treated rats in the stress or non-stress subgroup; (+) denotes *p* < 0.05, comparing rats to non-stressed, saline-treated rats in males or females; (+++) denotes *p* < 0.05, comparing rats to non-stressed, alcohol-treated rats in males or females

We investigated the effects of acute stress and adolescent alcohol exposure on the mRNA expression of genes related to the CRH signaling system in the amygdala and hypothalamus of male and female rats. These molecular results were also analyzed using three-way ANOVAs with sex (F_1_), stress (F_2_), and alcohol (F_3_) as factors (Fig. 2).

#### Amygdala

##### mRNA expression of *Crh*

The analysis of *Crh* expression in the amygdala revealed a significant main effect of alcohol [*F* [1,40] = 20.65, *p* < 0.001] (Fig. 2A), with alcohol-exposed rats showing higher mRNA levels of *Crh* compared to saline-treated rats. No other factors or interactions were associated with changes in *Crh* expression in the amygdala.

##### mRNA expression of *Crhr1*

There was a significant main effect of sex on *Crhr1* levels [*F* [1,40] = 6.82, *p* = 0.013], with female rats exhibiting lower *Crhr1* levels than male rats. Additionally, the analysis revealed a significant interaction between stress and alcohol [*F* [1,40] = 4.96, *p* = 0.032] (Fig. 2B). Specifically, alcohol-treated rats exhibited lower *Crhr1* levels compared to saline-treated rats within the non-stress subgroup, whereas no significant differences within the stress subgroup.

##### mRNA expression of *Crhr2*

For *Crhr2* expression in the amygdala, there was a significant main effect of stress on mRNA levels [*F* [1,40] = 18.90, *p* < 0.001], with stressed rats displaying lower *Crhr2* levels than non-stressed rats. Furthermore, a significant interaction between sex and alcohol was observed [*F* [1,40] = 5.52, *p* = 0.024] (Fig. 2C). While alcohol-treated rats had lower *Crhr2* levels compared to saline-treated rats in males, alcohol exposure did not affect this receptor in female rats.

#### Hypothalamus

##### mRNA expression of *Crh*

The analysis of *Crh* expression in the hypothalamus revealed significant main effects of sex and alcohol, along with a significant three-way interaction including sex, stress, and alcohol [*F* [1,40] = 5.94, *p* = 0.019] (Fig. 2D). To further explore this interaction, two-way ANOVAs with stress and alcohol were conducted separately for male and female rats: (a) In male rats, there were significant main effects of stress [*F* [1,20] = 9.03, *p* = 0.007] and alcohol [*F* [1,20] = 9.84, *p* = 0.005], with stressed and alcohol-treated rats displaying higher *Crh* levels than non-stressed and saline-treated rats, respectively. (b) In female rats, however, a significant interaction between stress and alcohol was observed [*F* [1,20] = 6.07, *p* = 0.023]. *Post hoc* comparisons revealed that alcohol-treated rats had higher *Crh* levels compared to saline-treated rats within the non-stress subgroup (*p* < 0.05). Additionally, stressed alcohol-treated rats exhibited lower *Crh* levels than non-stressed alcohol-treated rats (*p* < 0.05).

##### mRNA expression of *Crhr1*

There was a significant main effect of alcohol on mRNA levels of *Crhr1* and a significant interaction between sex and alcohol [*F* [1,40] = 19.77, *p* < 0.001] (Fig. 2E). Specifically, alcohol-treated female rats exhibited lower *Crhr1* compared to saline-treated females, whereas this effect was not statistically significant in male rats.

##### mRNA expression of *Crhr2*

 Regarding *Crhr2* expression in the hypothalamus, significant two-way interactions were observed (Fig. 2F). The interaction between sex and stress [*F* [1,40] = 6.42, *p* = 0.015] revealed higher *Crhr2* levels in stressed female rats compared to non-stressed female rats, primarily among alcohol-treated females; however, no significant differences were observed in male rats. As for the interaction between stress and alcohol [*F* [1,40] = 9.55, *p* = 0.004], alcohol-treated rats exhibited significantly lower *Crhr2* levels than saline-treated rats within the non-stress subgroup, but not within the stress subgroup.

### Effects of acute stress, adolescent alcohol, and sex on mRNA expression of NPY signaling-related genes in the brains of rats

**Fig. 3:**
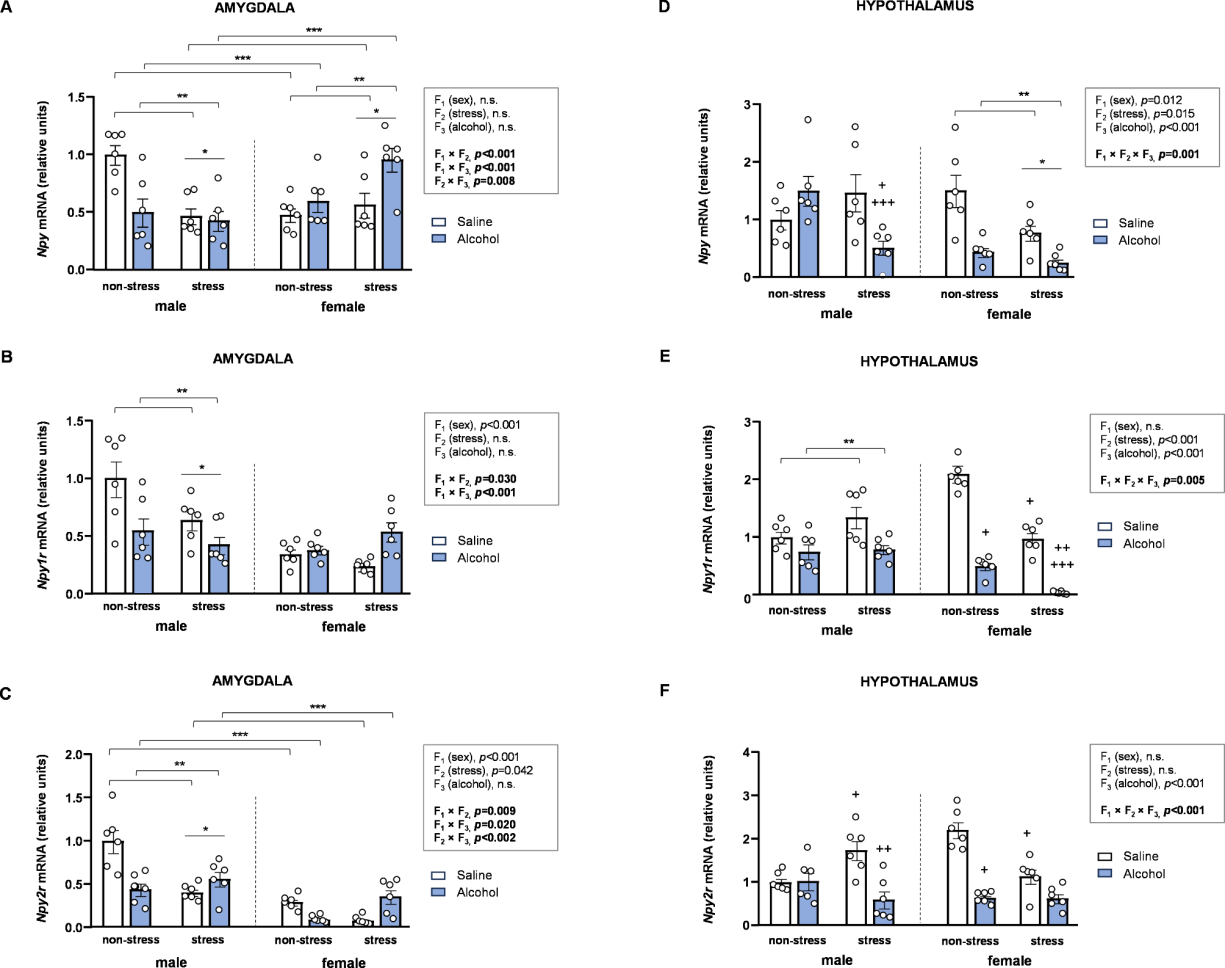
**Effects of acute stress**,** adolescent alcohol**,** and sex on NPY signaling-related genes in the amygdala and hypothalamus. ***Npy* expression in the amygdala **(A)**; *Npy1r* expression in the amygdala **(B)**; *Npy2r* expression in the amygdala **(C)**; *Npy* expression in the hypothalamus **(D)**; *Npy1r* expression in the hypothalamus **(E)**; and *Npy2r* expression in the hypothalamus **(F)** of male and female rats exposed to acute stress and alcohol exposure during adolescence. Bars represent the mean ± SEM (6 rats/subgroup). Data were analyzed using three-way ANOVA. Symbols are used to represent how significant interactions occur: (*) denotes *p* < 0.05, comparing stressed rats to non-stressed rats in males or females; (**) denotes *p* < 0.05, comparing alcohol-treated rats to saline-treated rats in males or females; (***) denotes *p* < 0.05, comparing alcohol-treated rats to saline-treated rats in the stress or non-stress subgroup; (+) denotes *p* < 0.05, comparing rats to non-stressed, saline-treated rats in males or females; (++) denotes *p* < 0.05, comparing rats to stressed, saline-treated rats in males or females; (+++) denotes *p* < 0.05, comparing rats to non-stressed, alcohol-treated rats in males or females

The mRNA expression of genes related to the NPY signaling system were also analyzed using three-way ANOVAs with sex (F_1_), stress (F_2_), and alcohol (F_3_) as factors (Fig. 3).

#### Amygdala

##### mRNA expression of *Npy*

Statistical analysis of *Npy* expression in the amygdala revealed significant two-way interactions (Fig. 3A). The interaction between sex and stress [*F* [1,40] = 16.24, *p* < 0.001] showed lower *Npy* levels in stressed male rats compared to non-stressed male rats; in contrast, stressed female rats exhibited higher *Npy* levels compared to non-stressed female rats. The interaction between sex and alcohol [*F* [1,40] = 16.20, *p* < 0.001] revealed lower *Npy* levels in alcohol-treated male rats compared to saline-treated male rats, whereas, alcohol-treated female rats had significantly higher *Npy* levels compared to saline-treated female rats. Regarding the interaction between stress and alcohol [*F* [1,40] = 7.93, *p* = 0.008], alcohol-treated rats exhibited lower *Npy* levels than saline-treated rats within the non-stress subgroup, while alcohol-treated rats showed higher *Npy* levels compared to their saline-treated counterparts within the stress subgroup. Taken together, these interactions together indicated the highest *Npy* levels in non-stressed, saline-treated male rats and stressed, alcohol-treated female rats.

##### mRNA expression of *Npy1r*

The analysis of *Npy1r* expression revealed a main effect of sex, along with notably significant two-way interactions between sex and other factors (Fig. 3B). The interaction between sex and stress [*F* [1,40] = 5.08, *p* = 0.030] showed lower *Npy1r* levels in stressed male rats compared to non-stressed male rats; however, no differences were observed in female rats. Similarly, the interaction between sex and alcohol [*F* [1,40] = 16.91, *p* < 0.001] revealed lower *Npy1r* levels in alcohol-treated male rats compared to saline-treated male rats, while no differences were observed in female rats.

##### mRNA expression of *Npy2r*

Regarding *Npy2r* in the amygdala, the three-way ANOVA revealed main effects of sex and stress, along with significant two-way interactions (Fig. 3C). The interaction between sex and stress [*F* [1,40] = 7.60, *p* = 0.009] showed lower *Npy2r* levels in stressed male rats compared to non-stressed male rats; however, no differences were observed in female rats. The interaction between sex and alcohol [*F* [1,40] = 5.86, *p* = 0.020] indicated that alcohol-treated male rats exhibited lower *Npy2r* levels compared to saline-treated male counterparts but no differences were observed in female rats. For the interaction between stress and alcohol [*F* [1,40] = 36.09, *p* < 0.001], alcohol-treated rats exhibited lower *Npy2r* levels compared to saline-treated rats within the non-stress subgroup, while alcohol-treated rats showed higher *Npy1r* levels than their saline-treated rats within the stress subgroup.

#### Hypothalamus

##### mRNA expression of *Npy*

The analysis of *Npy* expression in the hypothalamus revealed significant main effects of all three factors along with a significant three-way interaction [*F* [1,40] = 12.52, *p* = 0.001] (Fig. 3D). Two-way ANOVAs with stress and alcohol as factors were conducted separately by sex: (a) In male rats, a significant interaction between stress and alcohol was observed [*F* [1,20] = 9.89, *p* = 0.005]. *Post hoc* comparisons revealed higher *Npy* levels in alcohol-treated rats compared to saline-treated rats in the stress subgroup (*p* < 0.05), and lower *Npy* levels in stressed alcohol-treated rats compared to non-stressed alcohol-treated rats (*p* < 0.05). (b) In female rats, the analysis revealed significant main effects of stress [*F* [1,20] = 8.02, *p* = 0.010] and alcohol [*F* [1,20] = 23.64, *p* < 0.001], with stressed rats and alcohol-treated rats displaying lower *Npy* levels than non-stressed rats and saline-treated rats, respectively.

##### mRNA expression of *Npy1r*

Although there were significant main effects of stress and alcohol on *Npy1r* levels, a significant three-way interaction between sex, stress, and alcohol was observed [*F* [1,40] = 8.88, *p* = 0.005] (Fig. 3E). To further investigate, two-way ANOVAs with stress and alcohol were conducted separately in male and female rats: (a) In male rats, a significant main effect of alcohol was found [*F* [1,20] = 9.59, *p* = 0.006], with alcohol-treated rats displaying lower *Npy1r* levels compared to saline-treated rats. (b) In female rats, the analysis revealed a significant interaction between stress and alcohol [*F* [1,20] = 11.48, *p* = 0.003]. *Post hoc* comparisons showed lower *Npy1r* levels in alcohol-treated rats compared to saline-treated rats within both the non-stress (*p* < 0.05) and stress (*p* < 0.05) subgroups, as well as lower *Npy1r* levels in stressed rats compared to non-stressed rats (*p* < 0.05) within both the saline (*p* < 0.05) and alcohol (*p* < 0.05) subgroups.

##### mRNA expression of *Npy2r*

In this case, statistical analysis revealed a significant main effect of alcohol on *Npy2r* levels and a significant three-way interaction [*F* [1,40] = 23.85, *p* < 0.001] (Fig. 3F). Two-way ANOVAs with stress and alcohol as factors were conducted separately by sex: (a) In male rats, a significant interaction between stress and alcohol was found [*F* [1,20] = 10.16, *p* = 0.005]. *Post hoc* comparisons revealed lower *Npy2r* levels in alcohol-treated rats compared to saline-treated rats within the stress subgroup (*p* < 0.05), and higher *Npy2r* levels in stressed saline-treated rats compared to non-stressed saline-treated rats (*p* < 0.05). (b) In female rats, the analysis also revealed a significant interaction between stress and alcohol [*F* [1,20] = 15.18, *p* < 0.001]. *Post hoc* comparisons revealed lower *Npy2r* levels in alcohol-treated rats compared to saline-treated rats within the non-stress subgroup (*p* < 0.05), and higher *Npy2r* levels in stressed saline-treated rats compared to non-stressed saline-treated rats (*p* < 0.05).

### Effects of acute stress, adolescent alcohol, and sex on mRNA expression of corticoid signaling-related genes in the brains of rats

**Fig. 4:**
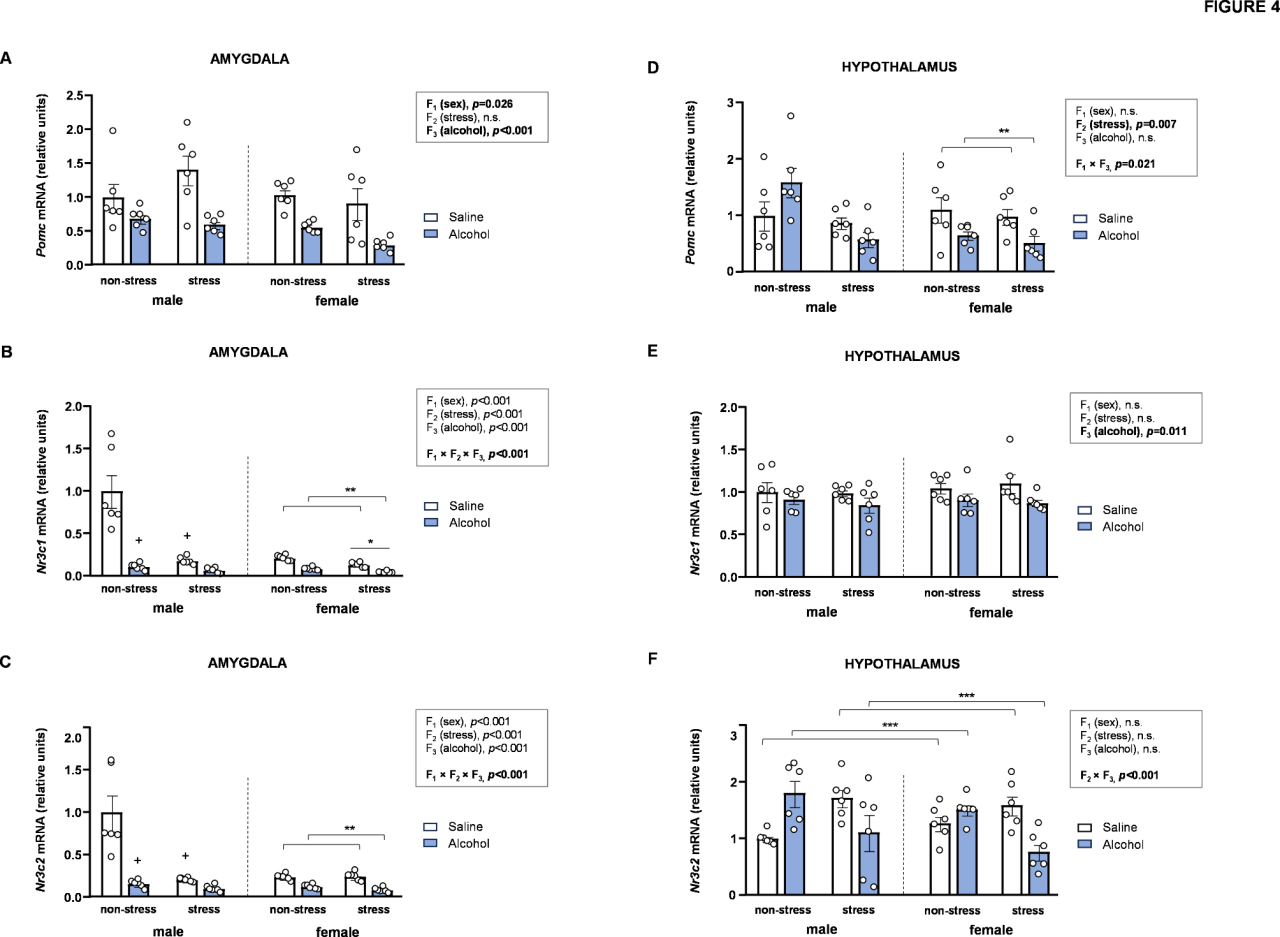
**Effects of acute stress**,** adolescent alcohol**,** and sex on corticoid signaling-related genes in the amygdala and hypothalamus. ***Pomc* expression in the amygdala **(A)**; *Nr3c1* expression in the amygdala **(B)**; *Nr3c2* expression in the amygdala **(C)**; *Pomc* expression in the hypothalamus **(D)**; *Nr3c1* expression in the hypothalamus **(E)**; and *Nr3c2* expression in the hypothalamus **(F)** of male and female rats exposed to acute stress and alcohol exposure during adolescence. Bars represent the mean ± SEM (6 rats/subgroup). Data were analyzed using three-way ANOVA. Symbols are used to represent how significant interactions occur: (*) denotes *p* < 0.05, comparing stressed rats to non-stressed rats in males or females; (**) denotes *p* < 0.05, comparing alcohol-treated rats to saline-treated rats in males or females; (***) denotes *p* < 0.05, comparing alcohol-treated rats to saline-treated rats in the stress or non-stress subgroup; (+) denotes *p* < 0.05, comparing rats to non-stressed, saline-treated rats in males or females

Given that both POMC and corticoid receptors are key components in regulating the HPA axis, the mRNA expression of *Pomc*, *Nr3c1*, and *Nr3c2* in the amygdala and hypothalamus were analyzed using three-way ANOVAs with sex (F_1_), stress (F_2_), and alcohol (F_3_) as factors (Fig. 4).

#### Amygdala

##### mRNA expression of *Pomc*

The analysis of *Pomc* expression in the amygdala revealed significant main effects of sex [*F* [1,40] = 5.32, *p* = 0.026] and alcohol [*F* [1,40] = 30.71, *p* < 0.001], indicating higher *Pomc* levels in male rats compared to female rats, and lower *Pomc* levels in alcohol-treated rats compared to saline-treated rats (Fig. 4A).

##### mRNA expression of *Nr3c1*

The gene expression of the glucocorticoid receptor (*Nr3c1*) was significantly affected by all factors, with a significant three-way interaction [*F* [1,40] = 14.49, *p* < 0.001] (Fig. 4B). Two-way ANOVAs with stress and alcohol as factors were conducted separately in male and female rats: (a) In male rats, a significant interaction between stress and alcohol was observed [*F* [1,20] = 16.27, *p* < 0.001]. *Post hoc* comparisons revealed lower *Nr3c1* levels in alcohol-treated rats compared to saline-treated rats within the non-stress subgroup (*p* < 0.05), and lower *Nr3c1* levels in stressed saline-treated rats compared to non-stressed saline-treated rats (*p* < 0.05). (b) In female rats, the analysis revealed significant main effects of stress [*F* [1,20] = 38.35, *p* < 0.001] and alcohol [*F* [1,20] = 118.30, *p* < 0.001], with lower *Nr3c1* levels observed in stressed rats compared to non-stressed rats, and in alcohol-treated rats compared to saline-treated rats.

##### mRNA expression of *Nr3c2*

Similar to *Nr3c1*, the analysis of *Nr3c2* mRNA expression revealed main effects of all factors, along with a significant three-way interaction [*F* [1,40] = 13.38, *p* < 0.001] (Fig. 4C). Two-way ANOVAs with stress and alcohol as factors revealed sex differences: (a) In male rats, a significant interaction between stress and alcohol was observed [*F* [1,20] = 12.02, *p* = 0.002]. *Post hoc* comparisons revealed lower *Nr3c2* levels in alcohol-treated rats compared to saline-treated rats within the non-stress subgroup (*p* < 0.05), and in stressed saline-treated rats compared to non-stressed saline-treated rats (*p* < 0.05). (b) In female rats, the analysis revealed significant a main effect of alcohol [*F* [1,20] = 85.45, *p* < 0.001], with lower *Nr3c2* levels observed in alcohol-treated rats compared to saline-treated rats.

#### Hypothalamus

##### mRNA expression of *Pomc*

Statistical analysis of *Pomc* expression revealed a significant main effect of stress [*F* [1,40] = 7.95, *p* = 0.007], with stressed rats showing lower *Pomc* levels than non-stresses rats (Fig. 4D). Additionally, a significant interaction between sex and alcohol was observed [*F* [1,40] = 5.81, *p* = 0.021]. Specifically, alcohol-treated female rats exhibited lower *Pomc* levels compared to alcohol-treated rats in females, while no differences were observed in male rats.

##### mRNA expression of *Nr3c1*

The analysis of *Nr3c1* expression in the hypothalamus revealed a significant main effect only of alcohol [*F* [1,40] = 7.18, *p* = 0.011] (Fig. 4E), indicating significantly lower *Nr3c1* levels in alcohol-treated rats compared to saline-treated rats.

##### mRNA expression of *Nr3c2*

Regarding the mineralocorticoid receptor, an interaction between stress and alcohol was observed in *Nr3c2* expression [*F* [1,40] = 24.45, *p* < 0.001] (Fig. 4F). Alcohol-treated rats had significantly higher *Nr3c2* levels than saline-treated rats within the non-stress subgroup; in contrast, alcohol-treated rats exhibited lower *Nr3c2* levels compared to saline-treated rats within the stress subgroup.

### Effects of acute stress, adolescent alcohol, and sex on mRNA expression of opioid receptor genes in the brains of rats

**Fig. 5:**
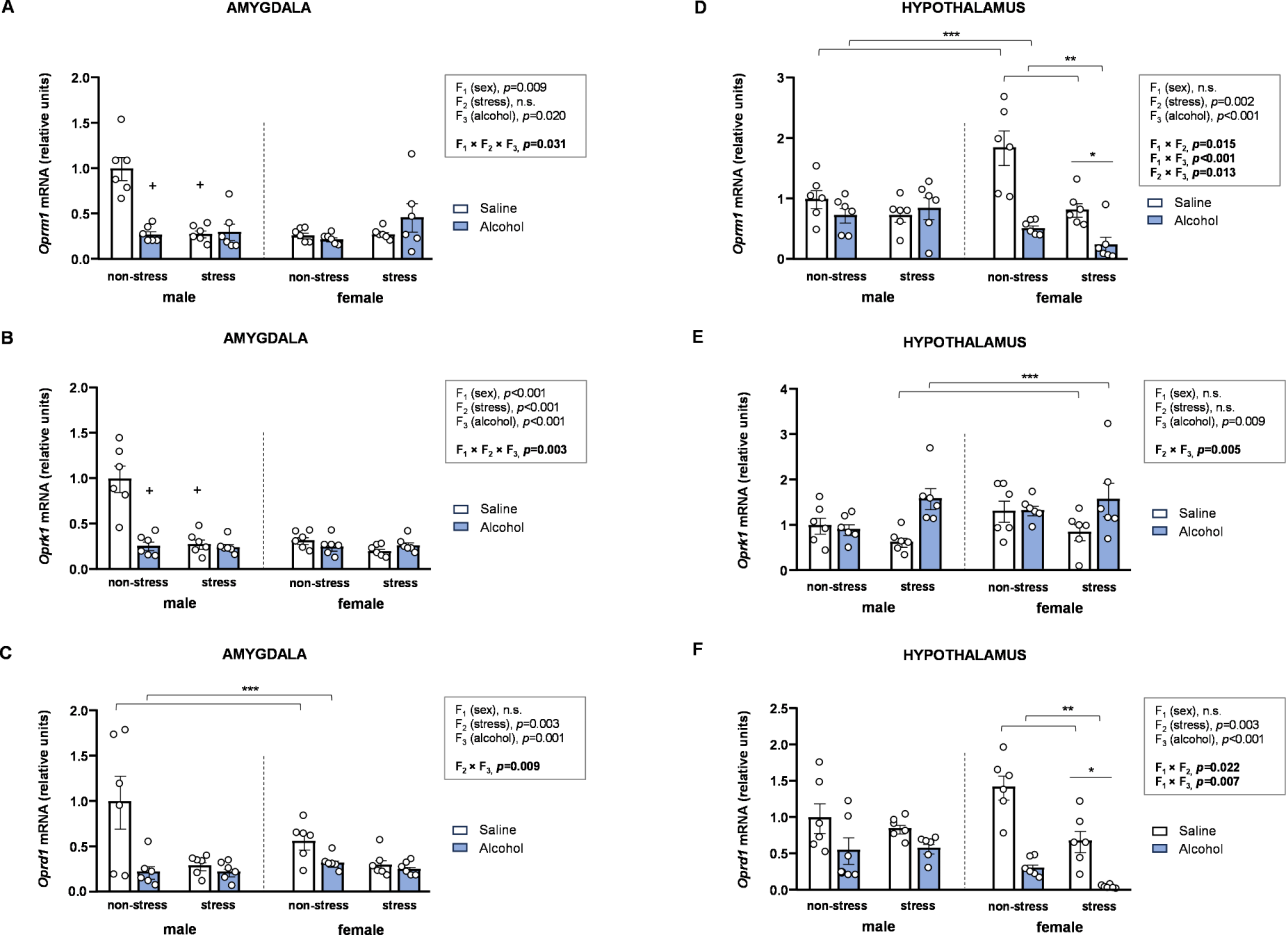
**Effects of acute stress**,** adolescent alcohol**,** and sex on opioid receptor genes in the amygdala and hypothalamus. ***Oprm1* expression in the amygdala **(A)**; *Oprk1* expression in the amygdala **(B)**; *Oprd1* expression in the amygdala **(C)**; *Oprm1* expression in the hypothalamus **(D)**; *Oprk1* expression in the hypothalamus **(E)**; and *Oprd1* expression in the hypothalamus **(F)** of male and female rats exposed to acute stress and alcohol exposure during adolescence. Bars represent the mean ± SEM (6 rats/subgroup). Data were analyzed using three-way ANOVA. Symbols are used to represent how significant interactions occur: (*) denotes *p* < 0.05, comparing stressed rats to non-stressed rats in males or females; (**) denotes *p* < 0.05, comparing alcohol-treated rats to saline-treated rats in males or females; (***) denotes *p* < 0.05, comparing alcohol-treated rats to saline-treated rats in the stress or non-stress subgroup; (+) denotes *p* < 0.05, comparing rats to non-stressed, saline-treated rats in males or females

The mRNA expression of key components of the opioid system was analyzed using three-way ANOVAs with sex (F_1_), stress (F_2_), and alcohol (F_3_) as factors (Fig. 5).

#### Amygdala

##### mRNA expression of *Oprm1*

The analysis of *Oprm1* expression in the amygdala revealed significant main effects of sex and alcohol, along with a significant three-way interaction [*F* [1,40] = 5.02, *p* = 0.031] (Fig. 5A). Two-way ANOVAs with stress and alcohol as factors were conducted in male and female rats to describe the three-way interaction: (a) In male rats, a significant interaction between stress and alcohol was observed [*F* [1,20] = 21.14, *p* < 0.001]. *Post hoc* comparisons showed lower *Oprm1* levels in alcohol-treated rats compared to saline-treated rats within the non-stress subgroup (*p* < 0.05), and lower *Oprm1* levels in stressed saline-treated rats compared to non-stressed saline-treated rats (*p* < 0.05). (b) In female rats, statistical analysis revealed no significant effects or interactions between stress and alcohol on *Nr3c2* levels.

##### mRNA expression of *Oprk1*

Although significant main effects of sex, stress, and alcohol on *Oprk1* expression were observed, a significant three-way interaction was also found [*F* [1,40] = 10.24, *p* = 0.003] (Fig. 5B). To further explore this interaction, two-way ANOVAs with stress and alcohol as factors were conducted separately by sex on *Oprk1* levels: (a) In male rats, a significant interaction between stress and alcohol was observed [*F* [1,20] = 18.19, *p* < 0.001]. *Post hoc* comparisons revealed lower *Oprk1* levels in alcohol-treated rats compared to saline-treated rats within the non-stress subgroup (*p* < 0.05), and lower *Oprk1* levels in stressed saline-treated rats compared to non-stressed saline-treated rats (*p* < 0.05). (b) In female rats, statistical analysis revealed no significant effects or interactions between stress and alcohol on *Oprk1* levels, similar to what was observed with *Oprm1*.

##### mRNA expression of *Oprd1*

Significant main effects of stress and alcohol on *Oprd1* expression were observed; however, a significant interaction between the two factors was also found [*F* [1,40] = 7.65, *p* = 0.009] (Fig. 5C). Specifically, alcohol-treated rats had significantly lower *Oprd1* levels than saline-treated rats within the non-stress subgroup, whereas no differences were found within the stress subgroup.

#### Hypothalamus

##### mRNA expression of *Oprm1*

Statistical analysis of *Oprm1* expression in the hypothalamus revealed significant main effects of stress and alcohol, along with two-way interactions involving sex, stress, and alcohol (Fig. 5D). The interaction between sex and stress [*F* [1,40] = 6.45, *p* = 0.015] revealed lower *Oprm1* levels in stressed female rats compared to non-stressed female rats; however, no differences were observed in male rats. The interaction between sex and alcohol [*F* [1,40] = 15.94, *p* < 0.001] showed significantly lower *Oprm1* levels in alcohol-treated female rats compared to saline-treated female rats, with no differences observed in male rats. Finally, the interaction between stress and alcohol [*F* [1,40] = 6.72, *p* = 0.013] revealed that alcohol-treated rats exhibited lower *Oprm1* levels than saline-treated rats within the non-stress subgroup, while this decrease did not reach significance within the stress subgroup.

##### mRNA expression of *Oprk1*

In addition to a significant main effect of alcohol on *Oprk1* expression, a significant interaction between stress and alcohol was observed [*F* [1,40] = 9.02, *p* = 0.005] (Fig. 5E). Specifically, the interaction revealed higher *Oprk1* levels in alcohol-treated rats compared to saline-treated rats within the stress subgroup, while no differences were observed within the non-stress subgroup.

##### mRNA expression of *Oprd1*

The analysis of *Oprd1* expression revealed significant main effects of stress and alcohol, along with two-way interactions involving all factors (Fig. 5F). The interaction between sex and stress [*F* [1,40] = 5.68, *p* = 0.022] showed lower *Oprd1* levels in stressed female rats compared to non-stressed female rats, with no differences in male rats. A second significant interaction between sex and alcohol [*F* [1,40] = 8.05, *p* = 0.007] showed significantly lower *Oprd1* levels in alcohol-treated female rats compared to saline-treated female rats, while these lower *Oprd1* levels did not reach significance in male rats.

### Effects of acute stress, adolescent alcohol, and sex on mRNA expression of opioid signaling-related genes in the brains of rats

The mRNA expression of other receptors associated with the opioid system was also analyzed using three-way ANOVAs.

#### Amygdala

##### mRNA expression of *Oprs1*

**Fig. 6:**
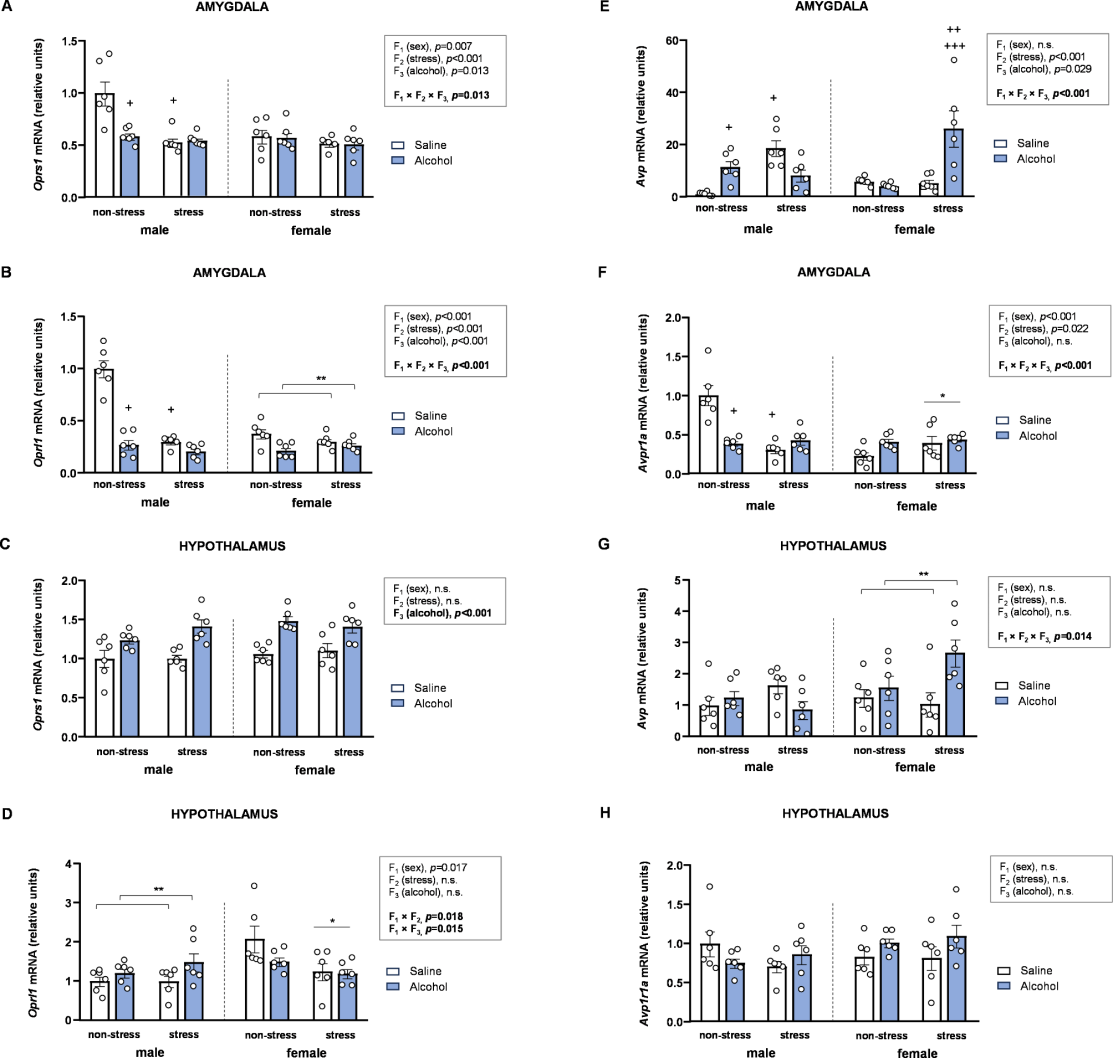
**Effects of acute stress**,** adolescent alcohol**,** and sex on opioid signaling-related genes and vasopressin system genes in the amygdala and hypothalamus. ***Oprs1* expression in the amygdala **(A)**; *Oprl1* expression in the amygdala **(B)**; *Oprs1* expression in the hypothalamus **(C)**; *Oprl1* expression in the hypothalamus **(D)**; *Avp* expression in the amygdala **(E)**; *Avpr1* expression in the amygdala **(F)**; *Avp* expression in the hypothalamus **(G)**; and *Avpr1* expression in the hypothalamus **(F)** of male and female rats exposed to acute stress and alcohol exposure during adolescence. Bars represent the mean ± SEM (6 rats/subgroup). Data were analyzed using three-way ANOVA. Symbols are used to represent how significant interactions occur: (*) denotes *p* < 0.05, comparing stressed rats to non-stressed rats in males or females; (**) denotes *p* < 0.05, comparing alcohol-treated rats to saline-treated rats in males or females; (+) denotes *p* < 0.05, comparing rats to non-stressed, saline-treated rats in males or females; (++) denotes *p* < 0.05, comparing rats to stressed, saline-treated rats in males or females; (+++) denotes *p* < 0.05, comparing rats to non-stressed, alcohol-treated rats in males or females

Statistical analysis of *Oprs1* expression revealed significant main effects of all factors, along with a significant three-way interaction [*F* [1,40] = 6.82, *p* = 0.013] (Fig. 6A). Once again, two-way ANOVAs with stress and alcohol as factors were conducted separately in male and female: (a) In male rats, a significant interaction between stress and alcohol was observed [*F* [1,20] = 11.24, *p* = 0.003]. *Post hoc* comparisons showed lower *Oprs1* levels in alcohol-treated rats compared to saline-treated rats within the non-stress subgroup (*p* < 0.05), and lower *Oprs1* levels in stressed saline-treated rats compared to their non-stressed saline-treated counterparts (*p* < 0.05). (b) In female rats, statistical analysis revealed no significant effects or interactions between stress and alcohol.

##### mRNA expression of *Oprl1*

The analysis of *Oprl1* expression in the amygdala showed significant main effects of all factors, along with a significant three-way interaction [*F* [1,40] = 18.84, *p* < 0.001] (Fig. 6B). Two-way ANOVAs with stress and alcohol as factors in male and female rats revealed the following: (a) In male rats, a significant interaction between stress and alcohol was observed [*F* [1,20] = 41.05, *p* < 0.001]. *Post hoc* comparisons revealed lower *Oprl1* levels in alcohol-treated rats compared to saline-treated rats within the non-stress subgroup (*p* < 0.05), and lower *Oprl1* levels in stressed saline-treated rats compared to non-stressed saline-treated rats (*p* < 0.05). (b) In female rats, statistical analysis revealed a significant main effect of alcohol on *Oprl1* levels [*F* [1,20] = 9.69, *p* = 0.006], with significantly lower *Oprl1* levels in alcohol-treated rats compared to saline-treated rats.

#### Hypothalamus

##### mRNA expression of *Oprs1*

The analysis of *Oprs1* expression showed a significant main effect of alcohol [*F* [1,40] = 43.97, *p* < 0.001], with alcohol-treated rats displaying higher *Oprs1* levels than saline-treated rats (Fig. 6C).

##### mRNA expression of *Oprl1*

Regarding *Oprl1* expression in the hypothalamus, statistical analysis revealed a significant main effect of sex and significant two-way interactions between sex and other factors (Fig. 6D). The interaction between sex and stress [*F* [1,40] = 6.50, *p* = 0.015] showed lower *Oprl1* levels in stressed female rats compared to non-stressed female rats, with no differences in male rats. Another significant interaction between sex and alcohol [*F* [1,40] = 6.08, *p* = 0.018] revealed significantly higher *Oprl1* levels in alcohol-treated male rats compared to saline-treated male rats, while no differences were observed in female rats.

### Effects of acute stress, adolescent alcohol, and sex on mRNA expression of vasopressin system genes in the brains of rats

The mRNA expression of *Avp* and *Avpr1a* was analyzed using three-way ANOVAs with sex (F_1_), stress (F_2_), and alcohol (F_3_) as factors.

#### Amygdala

##### mRNA expression of *Avp*

Statistical analysis of *Avp* expression in the amygdala showed significant main effects of stress and alcohol, along with a significant three-way interaction [*F* [1,40] = 27.10, *p* < 0.001] (Fig. 6E). To further explore the interaction, two-way ANOVAs with stress and alcohol as factors were conducted separately by sex: (a) In male rats, a significant interaction between stress and alcohol was observed [*F* [1,20] = 22.44, *p* < 0.001]. *Post hoc* comparisons revealed higher *Avp* levels in alcohol-treated rats compared to saline-treated rats within the non-stress subgroup (*p* < 0.05), and higher *Avp* levels in stressed saline-treated rats compared to non-stressed saline-treated rats (*p* < 0.05). (b) In female rats, a significant interaction between stress and alcohol was also observed [*F* [1,20] = 10.14, *p* = 0.005]. *Post hoc* comparisons revealed higher *Avp* levels in alcohol-treated rats compared to saline-treated rats within the stress subgroup (*p* < 0.05), and higher *Avp* levels in stressed alcohol-treated rats compared to non-stressed alcohol-treated rats (*p* < 0.05).

##### mRNA expression of *Avpr1a*

The analysis of *Avpr1a* expression revealed significant main effects of sex and stress, along with a significant three-way interaction [*F* [1,40] = 22.75, *p* < 0.001] (Fig. 6F). Two-way ANOVAs with stress and alcohol as factors was conducted in male and female rats: (a) In male rats, a significant interaction between stress and alcohol was observed [*F* [1,20] = 24.45, *p* < 0.001]. *Post hoc* comparisons revealed lower *Avpr1* levels in alcohol-treated rats compared to saline-treated rats within the non-stress subgroup (*p* < 0.05), and lower *Avpr1a* levels in stressed saline-treated rats compared to non-stressed saline-treated rats (*p* < 0.05). (b) In female rats, statistical analysis revealed a significant main effect of stress on *Avpr1a* levels [*F* [1,20] = 4.81, *p* = 0.040], with significantly higher *Avpr1a* levels in stressed rats compared to non-stressed rats.

#### Hypothalamus

##### mRNA expression of *Avp*

The analysis of *Avp* expression revealed a significant three-way interaction [*F* [1,40] = 6.55, *p* = 0.014] (Fig. 6G). Two-way ANOVAs with stress and alcohol as factors were conducted separately by sex: (a) In male rats, statistical analysis showed no significant effects or interactions between stress and alcohol. (b) In female rats, a significant main effect of alcohol was observed [*F* [1,20] = 6.80, *p* = 0.017], with alcohol-treated rats exhibiting higher *Avp* levels than saline-treated rats.

##### mRNA expression of *Avpr1a*

Regarding *Avpr1a* expression in the hypothalamus, the three-way ANOVA revealed no significant main effects or interactions of the factors (Fig. 6H).

## Discussion

Stressful experiences and alcohol consumption during critical periods of brain maturation, such as adolescence, are associated with maladaptive behaviors and an increased risk of developing AUD and psychiatric disorders in adulthood [2, 15]. Consistent with this, we have previously found that restraint stress and intermittent alcohol exposure during adolescence produce an anxious phenotype associated with alterations in distinct brain pathways in male rats [29, 30]. However, sexual dimorphism exists in the expression of neurochemical circuits involved in the regulation of reward and stress responses, as well as sex-specific differences in susceptibility to AUD and psychiatric disorders, the role of which remains unknown. To this end, we investigated the separate and combined effects of acute stress and intermittent alcohol exposure during adolescence on stress hormone levels linked to HPA axis activity and the expression of genes associated with reward and stress responses in the amygdala and hypothalamus of male and female Wistar rats in young adulthood.

The main findings in this study are summarized as follows: [1] BAC levels increased after the final alcohol administration in both sexes, but stressed male rats exhibited lower BAC levels compared to non-stressed males, an effect not observed in female rats; [2] Male rats gained significantly more weight than females, with no effects of stress or alcohol factors; [3] Stressed female rats showed higher ACTH levels compared to non-stressed female rats, with no significant changes in male rats; [4] Stress increased plasma CORT levels in male rats, while stressed, alcohol-treated female rats had lower CORT levels compared to non-stressed female rats; [5] CRH system: Alcohol exposure resulted in significantly increased *Crh* mRNA levels in the amygdala across both sexes, with no additional effects of stress or sex. Female rats had lower *Crhr1* levels in the amygdala, while alcohol reduced *Crhr2* levels in males but not females. Significant interactions among sex, stress, and alcohol were found in the hypothalamus, with distinct patterns between sexes; [6] NPY system: In the amygdala, stress reduced *Npy* and *Npy1r* levels in male rats but increased them in female rats. Alcohol decreased *Npy2r* levels in male rats, with varied effects in females. Similar sex-specific patterns were observed in the hypothalamus; [7] Corticoid system: Stress and alcohol had complex, sex-dependent effects on *Pomc*, *Nr3c1*, *Nr3c2*; [8] Opioid receptors: Stress and alcohol blunted the elevated expression of *Oprm1*, *Oprd1*, and *Oprk1* in the amygdala of males and the hypothalamus of females; and [9] Vasopressin system: Stress and alcohol interacted significantly to affect *Avp* and *Avpr1a* expression in the amygdala, with stronger effects in female rats. In the hypothalamus, alcohol increased *Avp* levels in female rats, with no significant effects in males.

These findings demonstrate that sex plays a crucial role in how stress and alcohol exposure influence hormones in the plasma and gene expression related to reward and stress signaling pathways in the amygdala and hypothalamus. The lasting effects were often more pronounced or varied in female rats, highlighting the importance of considering sex differences in research on stress and substance use.

In our study, the intermittent alcohol administration procedure elevated BAC levels in a binge-like manner, reaching levels greater than 80 mg/dL within one hour of administration in both male and female rats. However, a sex-based difference in BAC was observed, with male rats exhibiting higher levels than female rats. These findings are consistent with previous studies that have reported higher BAC levels in male rats compared to females at the 60-minute mark [37], supporting the notion that BAC is influenced by multiple factors, including sex [38]. Notably, we observed a significant interaction effect between sex and stress, which was evident in stressed male rats. Consistent with published data [30], stressed male rats exhibited lower BAC levels than non-stressed males, while no effects of stress on BAC were observed in females. Although the mechanisms underlying this effect are not fully understood, it may be attributed to sex-specific differences in alcohol pharmacokinetics and elimination [39, 40].

The HPA axis plays a central role in the physiological response to stress, primarily through the release of ACTH, which triggers the synthesis and release of glucocorticoids, such as CORT from the adrenal cortex in rodents. Overall, we found that female rats exhibited higher ACTH and CORT levels than male rats, indicating a sex-based difference in HPA axis activity, consistent with previous reports [41–43]. However, interactions between sex and the other two factors were specific to each hormone. For ACTH, significantly higher levels were observed in stressed females compared to males, with no effect of alcohol exposure. In contrast, stressed male rats exhibited higher CORT levels than non-stressed male rats, with no effect of alcohol, while a significant interaction between stress and alcohol was found in females. Specifically, alcohol increased CORT levels in non-stressed female rats, but this increase was blunted in stressed female rats. These findings support the existence of sex differences in the regulation of the HPA axis and its hormonal reactivity to stressors [41, 44]. Furthermore, the combination of stress and alcohol exposure during adolescence in female rats may induce persistent dysfunction of the HPA axis, potentially leading to maladaptive anxiety-like behaviors and distinct susceptibility to the effects of alcohol in adulthood. However, the lack of behavioral data is a key limitation of this study, preventing a precise assessment of the anxiety status of both sexes. Nevertheless, previous studies have reported that elevated CORT levels are associated with anxiogenic-like behaviors [30, 45, 46].

To investigate the neurobiological mechanisms underlying the dysregulation of the HPA axis, we examined the gene expression of relevant signaling systems involved in reward and stress responses in the amygdala and hypothalamus of male and female rats. These brain regions are critical components of the neurocircuitry that regulates emotions, motivation, and reward, making them particularly vulnerable to the effects of stress and alcohol exposure during adolescence. Consequently, similar to the HPA axis, sex-specific disruptions in the maturation of these brain regions during adolescence may result in persistent changes in emotional behaviors and heightened sensitivity to stress.

The CRH system plays a critical role in stress and addiction, with many studies describing sex differences in its regulation [for review see [47]]. Our results align with these observations, as sex-based differences were found in the gene expression of this system in the amygdala and hypothalamus. In male rats, adolescent alcohol exposure was associated with significant increases in *Crh* mRNA levels and decreases in its receptor mRNA levels, primarily in the amygdala. Although stress also affected the gene expression of the CRH system, this effect was less pronounced compared to that of alcohol in males. These data are consistent with an upregulation of the CRH signaling system, which may be linked to the anxiogenic-like behaviors observed in male rats subjected to the same experimental protocol, as previously reported [30]. In female rats, region-dependent effects were observed. Specifically, while the gene expression profile in the amygdala was similar to that in males, including significant effects of alcohol, interactions between stress and alcohol were evident in the hypothalamus. Non-stressed females exhibited a marked increase in *Crh* mRNA levels and decreases in *Crhr1* and *Crhr2* mRNA levels following alcohol exposure; however, these effects were blunted in females exposed to acute stress. The sex differences in the effects of stress and alcohol on CRH gene expression in the hypothalamus of females may be associated with a negative feedback mechanism of glucocorticoids in response to adolescent stressors [48, 49].

The NPY signaling system, which exerts anti-stress and anxiolytic effects primarily through its interaction with NPY1 and NPY2 receptors in the brain [for review see [50, 51]], also showed a complex interaction with sex, stress, and alcohol in both the amygdala and hypothalamus. Consistent with previous studies [29, 52], male rats exposed to either stress or alcohol showed a decrease in *Npy* mRNA levels and its receptors in the amygdala, but the combination of both factors did not lead to alterations distinct from those caused by each factor alone. In contrast, although these inhibitory effects of stress and alcohol were not observed in the hypothalamus, their combination induced a marked decrease in the gene expression of the NPY system in males, particularly in *Npy* and *Npyr2*. These findings suggest that the combination of adolescent stress and alcohol in males may attenuate the protective effects of NPY, potentially increasing vulnerability to maladaptive behaviors [50, 51]. In females, we found differences in gene expression of in both brain regions compared to males, with lower mRNA levels of *Npy*, *Npyr1*, and *Npyr2* in the amygdala and higher levels in the hypothalamus. However, these sex differences were influenced by stress and alcohol exposure. Specifically, both stress and alcohol induced a marked decrease in mRNA expression of the NPY system in the hypothalamus of females, and these decreases were further enhanced when both factors were combined. These findings suggest a downregulation of the NPY system in the hypothalamus of females when stress and alcohol are present together, which could affect the anti-stress and anxiolytic properties of this system.

To further explore the activity of the HPA axis and stress hormones, we investigated the gene expression of POMC and corticoid receptors. While *Pomc* mRNA levels were similar in the amygdala of both sexes, sex-based differences emerged in the hippocampus when combined with stress and alcohol exposure. Alcohol exposure was associated with a decrease in *Pomc* mRNA levels in the amygdala of males and females; however, this effect was not observed in the hypothalamus of males, where a decrease was linked to stress instead. Previous studies in male rodents have reported increased *Pomc* mRNA levels in the hypothalamus following alcohol exposure [53–55]. Thus, the increases in *Pomc* mRNA levels resulting from adolescent alcohol exposure may induce a compensatory effect that persists into young adulthood, which could account for the reduced or non-significant levels observed in both brain regions.

The gene expression of glucocorticoid and mineralocorticoid receptors revealed similar patterns across brain regions, with intricate interactions among sex, stress, and alcohol. Notably, male rats showed elevated *Nr3c1* and *Nr3c2* mRNA levels in the amygdala, which were reduced in those exposed to adolescent stress and/or alcohol. In contrast, female rats exhibited lower overall expression of corticoid receptors in the amygdala, with a specific decrease observed in response to alcohol. Unlike in the amygdala, no sex-related effects on these receptors were found in the hippocampus. Alcohol exposure induced a decrease in *Nr3c1* mRNA levels in both sexes, although this effect was observed only in stressed rats. The effects of sex and stress on corticoid receptor expression in animals not exposed to alcohol may be inversely related to the effects on CORT and ACTH, given that corticoid receptors mediate negative feedback on production of ACTH and, ultimately, CORT [49]. In conclusion, our results suggest that adolescent alcohol exposure interferes with the potential association between corticoid receptors and stress hormones, which is also affected by sexual dimorphism depending on the brain region.

The opioid signaling system is another key component in the regulation of reward and stress responses [56]. We assessed the primary receptors of this system (*Oprm1*,* Oprk1*, and *Oprd1*), as well as other related receptors (*Oprs1* and *Oprl1*), in the amygdala and hypothalamus. Similar to corticoid receptors, male rats exhibited higher mRNA levels of these receptors in the amygdala compared to females in the absence of stress or alcohol. Male rats exhibited a pattern where elevated mRNA levels were blunted following adolescent stress and/or alcohol exposure. However, female rats showed lower overall expression of opioid receptors in the amygdala, with no significant effects of stress or alcohol, except for *Oprl1* mRNA levels, which were reduced by alcohol exposure. These results suggest that the opioid system within the amygdala may be more sensitive to stress and alcohol effects in males than females. The decrease in opioid receptor expression observed in males may impair the ability to manage stress, potentially increasing anxiety and other stress-related disorders. Moreover, since opioid receptors in the amygdala are involved in the regulation of the reward system [57], reduced expression may impact limbic signaling pathways, contributing to maladaptive addiction-related behaviors. In the hypothalamus, differences among various receptors and the effects of interactions among factors were observed. The expression of *Oprm1* and *Oprd1* in the hypothalamus was primarily altered in female rats, with a decrease in their levels observed following alcohol exposure, an effect that was further enhanced when both stress and alcohol were present. In contrast to the other opioid receptors, no sex-based differences were found in *Oprs1* mRNA levels. Alcohol exposure induced an increase in *Oprs1* mRNA levels, which was not affected by stress.

Previous studies have reported the involvement of the vasopressin system in many alcohol-related behaviors, as well as in the regulation of stress and anxiety [58–60]. Consequently, we also examined the mRNA levels of *Avp* and *Avpr1a* in these animals. Once again, we found significant interaction effects and sex differences in the mRNA expression of these genes. In male rats, stress and/or alcohol exposure were associated with an increase in *Avp* mRNA levels in the amygdala, while *Avpr1a* mRNA levels were decreased in a similar manner. However, no significant effects were observed in the hypothalamus. In female rats, the most notable finding was a marked increase in *Avp* mRNA levels in those exposed to both stress and alcohol in the amygdala and hypothalamus. These results are consistent with previous studies reporting sex differences in the vasopressin system, including its upregulation in males [61–64]. Since amygdalar *Avp* is linked to anxiogenic behaviors [65, 66], the upregulation of the vasopressin signaling system may represent a vulnerability factor for developing anxiety-like behaviors.

## Perspectives and significance

This study underscores the critical role of sex in modulating the long-term effects of stress and alcohol exposure during adolescence on reward and stress responses. The findings reveal significant sex-specific differences in hormone levels and gene expression in the amygdala and hypothalamus of male and female rats, suggesting distinct neurobiological mechanisms that contribute to differential susceptibility to psychiatric disorders, including AUD. The results emphasize that adolescence is a sensitive period for brain development, during which exposure to stress and alcohol can induce persistent changes in the HPA axis and related systems, potentially leading to maladaptive behaviors and anxiety-like conditions in adulthood. These insights highlight the importance of incorporating sex-specific approaches in research on and treatment of AUD and stress-related disorders.

Furthermore, the research identifies several neurobiological pathways, including the CRH, NPY, corticoid, opioid, and vasopressin systems, that are differentially affected by stress and alcohol in males and females. These findings provide novel insights into the biological basis of sex differences in stress responses and addiction, suggesting potential targets for developing tailored therapeutic interventions. However, important limitations of the study should be noted, particularly the need for protein quantification in these brain regions (as post-transcriptional and translational regulation mechanisms may also be affected) and the evaluation of anxiety- and depression-like behaviors in these animals at adulthood. Nonetheless, by advancing the understanding of the complex interactions between stress, alcohol, and neurobiological pathways, this study reinforces the need for multifaceted strategies to prevent and manage psychiatric disorders and substance use, particularly through early interventions during adolescence.

## Electronic supplementary material

Below is the link to the electronic supplementary material.

Supplementary Material 1

## Data Availability

No datasets were generated or analysed during the current study.
