# Supplementary material for: Sex-dependent effects of acute stress and alcohol exposure during adolescence on mRNA expression of brain signaling systems involved in reward and stress responses in young adult rats
Source: Biol Sex Differ. 2024 Sep 26;15:75. doi: 10.1186/s13293-024-00649-5 (PMC11426001; doi:10.1186/s13293-024-00649-5)
Supplement: Supplementary file 1 — Supplementary Material 1 [file 13293_2024_649_MOESM1_ESM.docx]

**Table S1.** Primer references*

| Gene description | Assay ID | Ref Seq | Amplicon Length |
| --- | --- | --- | --- |
| *Actb* | Rn00667869_m1 | NM_031144.3 | 91 |
| *B2m* | Rn00560865_m1 | NM_012512.2 | 58 |
| *Npy* | Rn00561681_m1 | NM_012614.2 | 63 |
| *Npy1r* | Rn02769337_s1 | NM_001113357.1 | 98 |
| *Npy2r* | Rn00576733_s1 | NM_023968.1 | 65 |
| *Crh* | Rn01462137_m1 | NM_031019.1 | 112 |
| *Crhr1* | Rn00578611_m1 | XM_006247542.2 | 58 |
| *Crhr2* | Rn00575617_m1 | NM_022714.1 | 82 |
| *Oprm1* | Rn01430371_m1 | NM_001038597.2 | 64 |
| *Oprk1* | Rn01448892_m1 | NM_017167.2 | 66 |
| *Oprd1* | Rn00561699_m1 | NM_012617.1 | 70 |
| *Oprs1* | Rn00578590_m1 | NM_030996.1 | 73 |
| *Oprl1* | Rn00668206_g1 | NM_031569.3 | 90 |
| *Avp* | Rn00690189_g1 | NM_016992.2 | 78 |
| *Avpr1a* | Rn00583910_m1 | NM_053019.2 | 65 |
| *Pomc* | Rn00595020_m1 | NM_139326.2 | 92 |
| *Nr3c1* | Rn01405582_m1 | NM_012576.2 | 68 |
| *Nr3c2* | Rn00565562_m1 | NM_013131.1 | 79 |

(*) Primer sequence and information were obtained from the genome database of TaqMan ® Gene Expression Assays for mRNA analysis in [Rn] rat (Thermo Fisher, Waltham, MA, USA)
